# Supplementary material for: Stimulation of the Pro-Resolving Receptor Fpr2 Reverses Inflammatory Microglial Activity by Suppressing NFκB Activity
Source: Int J Mol Sci. 2023 Nov 6;24(21):15996. doi: 10.3390/ijms242115996 (PMC10649357; doi:10.3390/ijms242115996)
Supplement: Supplementary file 1 [file ijms-24-15996-s001.zip › ijms-2688456-supplementary.pdf]

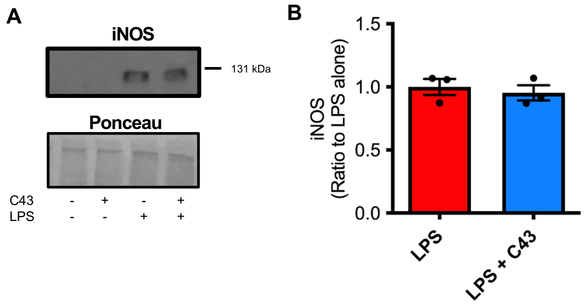

Supplementary Figure S1. Reduction of nitrite by C43 is independent of iNOS regulation. A, B) administration of 50 ng/ml LPS for 24 h upregulated the translation of iNOS compared to untreated, whereas 100 nM C43 had no effect on iNOS expression when administered either alone, or 1 h post-LPS. Data are means ± SEM of 3 independent cultures.

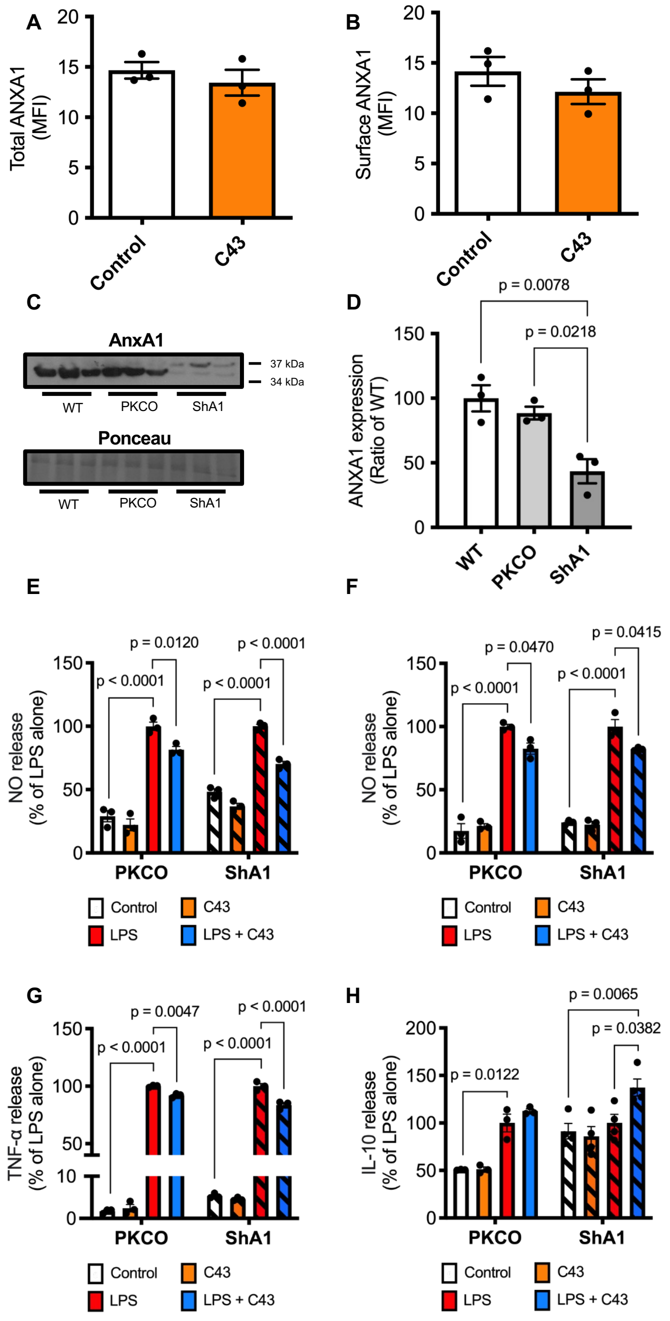

Supplementary Figure S 2: The anti-inflammatory effects of C43 are independent of ANXA1 regulation. A, B) the relative expression levels of both total and surface annexin A1 after 24 h treatment with 100 nM C43, determined with flow cytometry and 0.1% TX-100 cell permeabilization. **C**, **D**) transfection with ShA1 successfully reduced annexin A1 expression in BV2 microglia compared to untransfected and control plasmid transfection (PKCO). **E, F**; the anti-inflammatory nitrite reducing effects of 1 h post-treatment of C43 were retained even in PKCO and ShA1 transfected cells at 24 h (E) and 48 h (F). **G, H**; the effects of C43 on TNFα and IL-10 release at 48 h post-LPS administration were also retained in plasmid transfected microglia. Data are means ± SEM of 3-6 independent cultures, plated in triplicate.

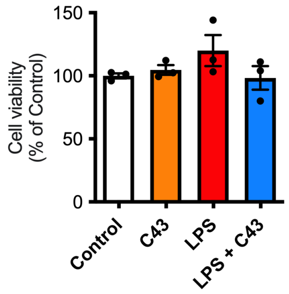

Supplementary Figure S3. Neither C43 nor LPS treatment, nor their combination, affected BV2 cell proliferation. Neither treatment of BV2 cells for 48 h with either 100 nM C43, 50 ng/ml LPS nor their combination (C43 administered 1 h post-LPS) affected cell numbers, as judged using the Prestoblue cell viability assay. Data are means ± SEM of 3 independent cultures.

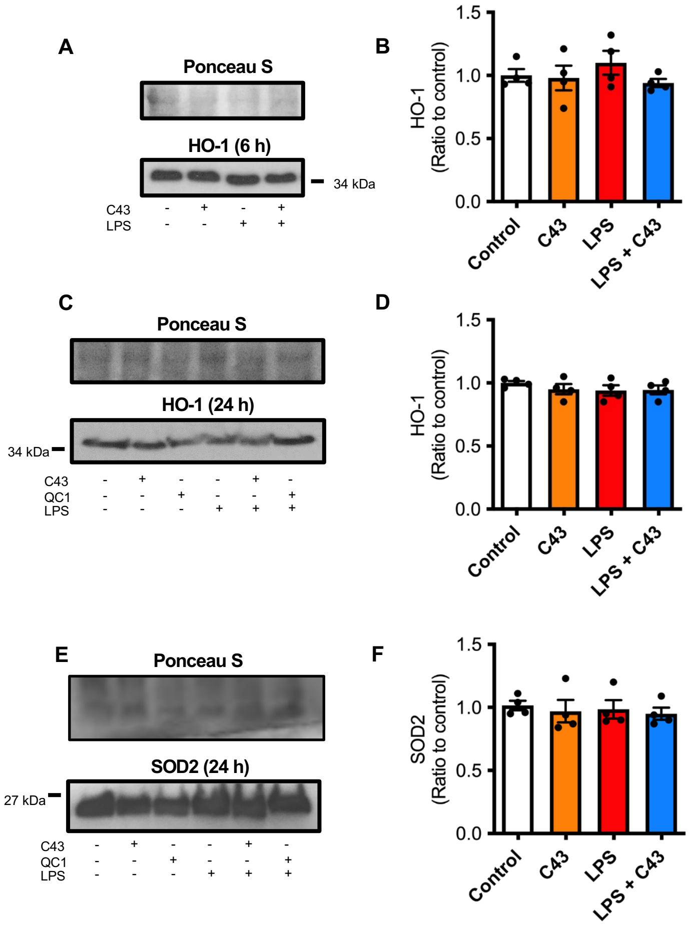

Supplementary Figure S 4: Neither LPS nor C43 treatment affected two major cellular antioxidant enzymes. A-D) no difference in HO-1 expression after 6 h (A, B) or 24 h (C, D) exposure to LPS was observed in BV2 microglia, with and without the 1 h post treatment of C43. **E, F**) SOD2 expression remained unchanged 24 h after LPS (50 ng/ml) addition, with 100nM C43 treatment (or the alternative Fpr2 agonist QC1, 100 nM) also unable to modulate protein expression. Western blot analysis is representative of 3 independent cultures, with samples normalized to Ponceau S-defined total protein content; densitometric analysis data are means ± SEM of 3 independent cultures.

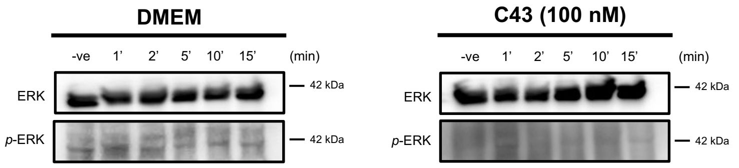

Supplemental Figure S5: C43 binding to Fpr2 has no short-term effect on ERK1/2 MAP kinase phosphorylation. Addition of C43 (100 nM) had no effect on ERK1/2 phosphorylation over a 15 minute time course, analogous to that used in studies of p38 MAP kinase activity. Data are representative of three independent cultures.
